# Do overqualified employees hide knowledge? The mediating role of psychological contract breach

**DOI:** 10.3389/fpsyg.2022.842725

**Published:** 2022-09-05

**Authors:** Huiqin Zhang, Linzhen Li, Xuanming Shan, Anhang Chen

**Affiliations:** College of Management Science, Chengdu University of Technology, Chengdu, China

**Keywords:** perceived overqualification, psychological contract breach, knowledge hiding, leader-member exchange, non-linear relationship

## Abstract

Although the negative effects of a sense of overqualification on organizations and individuals have been examined, it is debatable whether overqualified employees hide knowledge. Relying on the social comparison theory and psychological contract theory, this paper tried to investigate the non-linear relationship between perceived overqualification and knowledge hiding *via* psychological contract breach by surveying employees with bachelor’s degrees or above and eventually recruited 475 participants. The results indicated that psychological contract breach acts a partial mediating role in the inverted U-shaped relationship between perceived overqualification and knowledge hiding, while leader-member exchange acts as a moderator. The finding manifests that overqualification encourages employees to hide knowledge, but their possibly vigorous aspects will be displayed when a certain limit is exceeded. This study benefits organizations by advising them to dialectically treat and properly place overqualified employees and contributes to the research on overqualified employees’ knowledge management by offering a new explanation and complete understanding of perceived overqualification and knowledge hiding, with specific focus given to the psychological states of employees.

## Introduction

Faced with an increasingly complex and uncertain global business development environment, organizations need to innovate in products and services to occupy a place in the market (Fong et al., 2018). Selecting the right employees for organizations (Straatmann et al., 2020) and being aware of the value of employees’ knowledge (Ma and Zhang, 2021) is becoming particularly and increasingly vital. However, enterprises are more inclined to recruit employees with high knowledge and ability, so as to achieve the matching between employees and organizations, and hope that they can share knowledge (Ma and Zhang, 2021) to maximize the talent effect and promote organizational innovation while doing their job well. But it can lead to a large number of employees feeling overqualified, which has become an increasingly prevalent phenomenon around the world, and has aroused continuous attention from the businesses and academic community (Zhang et al., 2021a,b). *Perceived overqualified* (PO) has a variety of negative effects on organizations and individuals (Erdogan and Bauer, 2021), so that employees seem unlikely to share knowledge. However, do employees who feel overqualified necessarily hide their knowledge?

PO refers to employees who feel that their abilities exceed the needs of the position (Maynard et al., 2006). Available literature has discovered the passive influence of PO on enterprises and staff. For instance, PO leads to poor job satisfaction (Harari et al., 2017), decreasing extra-role behaviors (Erdogan et al., 2020), and increasing turnover intention (Maynard et al., 2006), counterproductive work behavior (Liu et al., 2015; Fine and Edward, 2017), and emotional exhaustion (Yu et al., 2019). However, its positive effects have also been found (Zhang et al., 2021b, etc.), such as job engagement and performance (Ma et al., 2020; Erdogan and Bauer, 2021). In terms of these inconsistent findings, some studies attested that, owing to different conditions, such as interpersonal relationships (Deng et al., 2018) and development-oriented organizational culture (Zhang et al., 2021b), PO’s effect is comparatively disparate. However, less attention has been paid to *knowledge hiding* (KH). KH has become prevalent in organizations, and has become an important obstacle to organizational innovation and growth (Anser et al., 2021). Present literature has found that the antecedents of KH chiefly contain individual ingredients [like psychological ownership (Huo et al., 2016), interpersonal trust (Yao et al., 2020), shared goals (Nadeem et al., 2021), job autonomy (Peng et al., 2021), and work alienation (Guo et al., 2021)], the relationships between colleagues [such as workplace ostracism (Zhao et al., 2016)], leadership styles (Oubrich et al., 2021; such as ethical leadership (Abdullah et al., 2019; Men et al., 2020; Anser et al., 2021) and abusive supervision (Ghani et al., 2020; Wang et al., 2021)), and organizational environment [such as competitive environment (Jha and Varkkey, 2018), organizational rewards (Zhang and Min, 2021), and organizational injustice (Jahanzeb et al., 2021)], along with failure to sufficiently focus on PO (Ma and Zhang, 2021). It is necessary to pay attention to whether and how overqualified employees carry out KH. Although Li et al. (2021) have confirmed the positive impact of PO on KH, Ma and Zhang (2021) have found its “U” relationship. And the relationship between PO and KH seems to be ambiguous. Therefore, it is indispensable to further explore the relation between PO and KH.

Discussion on the important mechanism of the relationship between PO and KH is comparatively rare. Employees with overperceived qualifications tend to have negative effects on organizations (Erdogan and Bauer, 2021), and the relatively intuitive one is the contract problem between employees and organizations (Luksyte et al., 2011). Employees feel overqualified for the job requirements [as the definition of PO (Maynard et al., 2006)], and it means that the organization does not offer appropriate positions to employees, breaking the tacit understanding between employees and the organization (Luksyte et al., 2011). The *breach of psychological contract* (PCB) can encourage negative behaviors of employees, such as employee workplace deviance (Chiu and Peng, 2008) and counterproductive work bearing (Bari et al., 2020a). We reasonably infer that PCB can lead to KH (Ghani et al., 2020). Moreover, existing studies pay more attention to discussing the relationship between PO and KH from the perspective of emotion (Li et al., 2021; Ma and Zhang, 2021), which may ignore cognitive perspectives to some extent (e.g., psychological contract breach). To sum up, in order to understand the transmission mechanism between PO and KH more comprehensively, this paper will explore the mediating role of PCB between PO and KH.

Additionally, there is a close relationship between employees’ KH and their affinities to their leaders (He et al., 2021; i.e., *leader-member exchange*, LMX). LMX defines the degree of contact between the employee and their leadership (Major and Morganson, 2011). The quality of the relationship with the leader will affect overqualified employees’ behaviors. A high-quality LMX is more likely to promote the negative behavior of overqualified employees, because the mismatch between qualifications and positions runs counter to employees’ previous expectations (Yu et al., 2019). However, under low-quality LMX, the relationship between overqualified employees and KH may be not consistent. As “outsiders,” they are prone to negative attitudes and behaviors, which bring negative impacts (Martin et al., 2016) to individuals and organizations (such as KH). But when the PO of employees has far exceeded those of their colleagues, they may reduce KH and tend to maintain the relationship with colleagues. It seems that, under different quality LMX, the influence of PO on KH will be different. Therefore, it is reasonable to further examine the moderating effect of leader-member exchange on the relationship between PO and KH.

**Figure 1:**
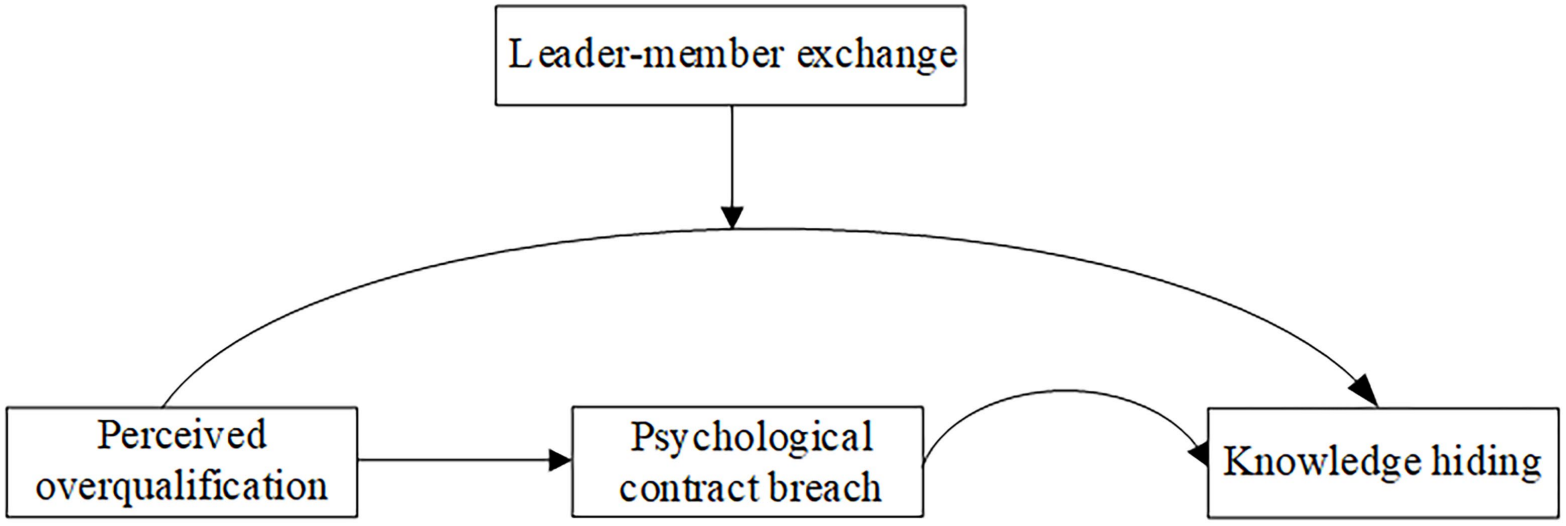
Study model.

Based on the social comparison theory (Buunk and Gibbons, 2007; Gerber et al., 2018), overqualified employees will compare themselves with employees with similar status or ideas (such as colleagues), resulting in knowledge hiding. However, when the knowledge, skills, and other qualifications of the employees have reached a certain level and are far more than their colleagues, they will reduce KH. Moreover, according to psychological contract theory, overqualification of employees will cause PCB which induces negative behaviors of employees, such as KH. Finally, we examined the strength of the relationship between PO and KH at the high and low levels of LMX. To sum up, the study model is displayed in Figure 1.

Our research contributed to current literature in the following three aspects. Firstly, at present, little attention has been paid to PO among the antecedents of knowledge hiding. And there are inconsistent findings in the available literature discussion on the relationship between PO and KH (Li et al., 2021; Ma and Zhang, 2021). On this basis, we further explore the relationship between PO and KH. The results provide new insights into the relationship between PO and KH and enrich the literature of PO and KH. Secondly, existing literature mainly emphasized the important role of emotion in the relationship between PO and KH (Ma and Zhang, 2021). Our study makes a new supplement to this proposition by studying the mediating role of psychological contract breach. From a cognitive perspective, we explain whether and why PO affects knowledge hiding, expanding and enriching the literature on the mechanism of knowledge management. Thirdly, taking LMX as a moderating variable, we explain the boundary conditions of PO affecting KH, providing corresponding suggestions for managers to reduce knowledge hiding.

## Theoretical background and hypotheses

### Perceived overqualification and knowledge hiding

Perceived overqualification (PO) means that an individual’s education level, knowledge, experience, and skills are higher than that required by the post (Maynard et al., 2006). It has been proved that PO has a strong negative predictive effect (Erdogan and Bauer, 2021). According to social comparison theory, there is a comparison between employees and their coworkers based on abilities and attitudes, which contributes to employees’ self-evaluation to obtain self-cognition (Buunk and Gibbons, 2007; Gerber et al., 2018).

According to this theory, the motivation for employees to hide their knowledge lies in their competitive advantage or ability when compared with others. Under the condition of low and middle PO, the ability of employees is only a little more than the job requirements, so employees have a strong sense of crisis and fear that their colleagues will surpass them. Therefore, they will deliberately hide their knowledge to speed up their self-improvement and gain competitive advantages (Li et al., 2021). But overqualified employees do not necessarily hide knowledge. If PO reaches a certain level, it means that the knowledge, skills, and other qualifications of overqualified employees exceed their job requirements and accumulated to a certain extent. At this time, the motivation of overqualified employees to hide knowledge is the strongest, and their knowledge hiding will reach the highest point. As PO increases, overqualified employees perceive that their abilities are far beyond the job requirements. For one thing, such intense underemployment will make employees change their work content to match their needs and aspirations (Lin et al., 2017). For another, the strong perception of superiority of employees leads to greater satisfaction in their demand of respect and recognition, and they will have higher psychological security and are more willing to participate in teamwork and share their knowledge (Lin et al., 2017). Their focus shifted from comparison with colleagues to knowledge interaction, and the motivation of KH gradually declined. In conclusion, with the increase of PO, KH should rise first and then decline. Thus, we consider that PO and KH have an inverted U-shaped, relationship and propose the below assertion:

*H1*: PO and KH have an inverted U-shaped relationship. In the low and middle (middle and high) level of PO, PO is positively (negatively) related with KH.

### The mediating effect of psychological contract breach

Psychological contract breach (PCB) is an informal agreement within an organization and it refers to employees’ cognition caused by the enterprises’ failure to perform its promise (Robinson and Morrison, 2000). Another concept closely related to PCB is psychological contract violation, and it is easy to get confused between the two. But there are differences between these two concepts. PCB is a cognitive evaluation, while psychological contract violation mainly means emotional experience, and the former can affect the latter (Morrison and Robinson, 1997). Existing research has found that PCB is influenced by a variety of factors, such as employees’ psychological perception (Ma et al., 2019) and managers’ behaviors (Ghani et al., 2020). Generally, psychological contract theory is used to comprehend the mutual relationship between individuals and organizations, which is manifested as a psychological tacit understanding (Kutaula et al., 2020). Overqualified employees have higher knowledge, skills, and abilities, and are more looking forward to career development and promotion space to alleviate their PO. They expect the organization to provide them with a position that can give full play to their abilities and also provide development space, so as to determine their attitudes and behaviors toward the organization. Therefore, overqualified employees contend that the organization has violated the promise made to them at the time of recruitment so that they lose matching positions, and this then leads to their PCB.

*H2*: PO is rightly associated with PCB.

PCB can lead to a series of negative behaviors. The breach of psychological contract will facilitate counterproductive workplace behavior (Li and Chen, 2018) and negative effects on employees’ job satisfaction (Hartmann and Rutherford, 2015; Ampofo, 2020) and organizational identification (Tufan and Wendt, 2020). Similarly, PCB can also promote knowledge hiding (Ghani et al., 2020). On account of the psychological contract theory, there is a psychological contract between employees and the organization. In such a tacit environment, employees have certain expectations for the organization and perceive their relationship with the organization in this way (Kutaula et al., 2020). When the mental contract between employees and organizations is broken, employees will gradually become dissatisfied with the organization and keep silent (Bari et al., 2020b) in the knowledge interaction to eliminate the psychological imbalance. However, the relation between PCB and KH is not invariable. Some studies show that, in the case of certain circumstances, the negative effects of psychological contract breaking will be weakened, and even positive effects will be produced. For instance, there is a subtle contract between PCB and individual ‘s citizenship behaviors (Gupta et al., 2016) and life satisfaction (Ampofo, 2020). Moreover, Griep and Vantilborgh (2018) confirmed that PCB may promote citizenship behaviors although it seems ambivalent. We can reasonably infer that PCB can promote KH. When PCB reaches a certain level, employees’ focus on KH will shift. In the short-term, PCB can lead to KH, but in the long-term, the negative effects of a broken psychological contract may dissipate, that is, they may be immediate and short-lived (Vantilborgh et al., 2016). Especially for young people, they see it more as a challenge than an obstacle (Vantilborgh et al., 2016). And although employees have PCB, they value collective interests more than individual interests (Jackson et al., 2006), particularly in China. Competent employees are more likely to take responsibility and contribute to the organization, and they are less likely to hide their knowledge. And when overqualified employees share the same goals with their colleagues and feel supported by them, the negative impacts between PCB and outcomes will be alleviated (Ng et al., 2014) and are less likely to increase KH in knowledge interaction. Therefore, as the PCB continues to increase, the motivation of employees to hide knowledge from colleagues will weaken, and the KH will decrease, although employees are in the middle and high degree of PCB. In conclusion, with the increase of PCB, KH should rise first and then decline, and there is a reverse U-shaped connection between PCB and KH.

*H3*: There is an inverted U-shaped relation between PCB and KH.

In conclusion, employees’ PO can promote their PCB, which has a reverse U-shaped relationship to KH. In the condition of low and medium levels of PO, according to psychological contract theory, employees deem that the agreement between them and the organization has been broken, the degree of PCB of employees becomes stronger, and their KH gradually increases. In terms of high and medium levels of PO, the degree of PCB of employees becomes stronger. But their intention to hide knowledge is weak, because employees, especially those with comparatively high abilities, will pay more attention to the collective interests, and the KH of employees gradually decreases. Therefore, we suggest as follows:

*H4*: PCB mediates the inverted U-shaped relation between employee PO and KH.

### The moderating effect of leader-member exchange

The differences of KH among different overqualified employees may be related to the interaction between employees and their leaders (He et al., 2021). Leader-member exchange (LMX) stands for the degree of contact between the employee and the leader (Major and Morganson, 2011). Due to the limitation of time and energy and the differentiated personality characteristics of subordinates, leaders will adopt differentiated management styles (Kong et al., 2019). Employees who were considered “insiders” had closer contact with their leaders, which led to more resources, trust, and privilege (Zhao et al., 2019), such as more pay, promotion opportunities, and job autonomy. Existing literature mainly discusses the positive aspects of LMX; for instance, high-quality LMX can promote employees’ performance (Martin et al., 2016), creativity (Kong et al., 2019), and knowledge sharing (Choi et al., 2019; Kim et al., 2021) and relieves employees’ social comparison tension (Matta and Van Dyne, 2020) and cyberloafing (Usman et al., 2021), but less attention is paid to its negative side. However, giving more resources and privileges to subordinates does not necessarily lead to positive results, but can sometimes be self-defeating. For highly qualified employees who perceive excess qualifications, they have higher expectations of the organization, and when the gap between subjective and objective expectations is too large, it is more likely to stimulate negative behavior (Yu et al., 2019). They may feel a sense of betrayal (Restubog et al., 2010), thus promoting the emergence of knowledge-hiding behavior.

On the contrary, staff with low-quality LMX acquire limited resources and are usually confined to normal working relationships (Kong et al., 2019). In the case of low-quality LMX, employees with low or medium PO are regarded as “outsiders” and find it difficult to get resources and attention from leaders. Employees may feel a stronger sense of unrewarded ambition than in high-quality LMX and experience negative emotions such as boredom (Sanchez-Cardona et al., 2020), anger, and complacency (such as low organizational self-esteem, etc.; Liu and Wang, 2012). Employees will choose to hide their knowledge to alleviate psychological imbalance (van Dijk et al., 2020). But, in the condition of bad interaction with leaders, the high level of PO’s employees are more likely to get along well with colleagues. Employees are gradually recognized by colleagues (van Dijk et al., 2020) and perceive that they are accepted and understood by their colleagues, so they are more willing to participate in team cooperation and information sharing (Buunk and Gibbons, 2007), and their KH decreases accordingly. Therefore, we put forward the following hypothesis:

*H5*: LMX moderated the reverse U-shaped relationship between PO and KH. Compared with high-quality LMX, the positive and negative relationships between PO and KH were stronger in low-quality LMX.

## Materials and methods

### Samples and procedure

At present, the phenomenon of overqualification of employees is very serious in China (Zhang et al., 2021a), especially in the manufacturing industry, IT/Internet industry, and financial industry. And in view of the fact that PO and KH usually occur in knowledge-intensive industries (He et al., 2021) and employees with a comparatively high knowledge reserve (Ma and Zhang, 2021), employees mainly from the industry of manufacturing, IT/Internet, and finance in China with a bachelor’s degree or above are selected in this study. In consideration of the actual needs of hypothesis verification and variable measurement involved in this study, random sampling was surveyed by using an online questionnaire. And to assure that high-quality data can be collected professionally, a professional online questionnaire survey platform was employed. The research company used in this study is a professional and authoritative questionnaire survey platform in China, which has a large number of market users and is frequently used and favored by researchers. In total, 524 questionnaires were received and 475 valid ones were obtained with a valid recovery of 90.65% after deleting invalid questionnaires. Our sample size met the minimum requirement of no less than 200 samples required for four-indicator model test in the conventional mode, and as many samples as possible were collected (Kim, 2012). And the effective response rate reached 90%, which well addresses the problem of non-response bias (Sedgwick, 2014). Table 1 is the basic information of the research sample.

**Table 1:** Basic sample information.

Variable	Classify	Proportion (%)	Variable	Classify	Proportion (%)
Gender	Male	37.50	Tenure	1 year or less	6.3
	Female	62.50		2 to 3 years	26.5
Age	20 to 25	10.105		4 to 5 years	30.9
	26 to 30	46.105		6 to 10 years	29.1
	31 to 35	30.53	Position	10 years and above	7.2
	36 to 40	8.63		Ordinary employees	36.0
	Above 40	4.63		Low-level managers	40.0
Education	Undergraduate	78.7		Middle management	22.1
	Master	19.8		Senior management	1.9
	Doctor	1.5	Company nature	State-owned enterprises	32.8
				Private enterprise	67.2

### Measures

The scale applied to this study is relatively mature, and necessary adjustments and modifications are made to some items of the scale according to the actual situation. All scales were measured by Likert five-point scoring method.

*Perceived overqualification* has adopted the scale of Maynard et al. (2006), consisting of 9 items. Based on the research needs of localization in China, this study selects five statements, such as “my education level is higher than the job requirements.” Its Cronbach’s α coefficient is 0.751.

*Knowledge hiding* was measured with a three-dimensional scale adopted by Connelly et al. (2012), including 12 items. Because of disdain for colleagues, overqualified employees may engage in evasive hiding or play dumb, but few of them engage in rationalized hiding (Li et al., 2021), so this paper does not explore rationalized hiding. Based on the research needs of localization in China, seven questions were selected from two dimensions, including evasive hiding (three questions) and playing dumb (four questions). Taking “I may pretend that I do not know this knowledge” as an example (α = 0.871).

*Psychological contract breach* employed the scale used by Robinson and Morrison (2000), with five items. For instance, “I did not receive the return I was promised for my contributions” (α = 0.895).

*Leader-member exchange* referred to the 7-item tool of Graen and Uhl-Bien (1995). Based on localization requirements, this paper selected six statements, such as “I know how I get on with my leader and whether my leader is satisfied with my work performance” (α = 0.814).

Referring to previous studies, we took the gender, age, education, tenure, position, and company nature of employees as control variables (Yao et al., 2020; Mubarak et al., 2021).

## Results

### Common method bias

Self-reporting is an important method in organizational behavior investigation which needs to deal with method bias problem (Yao and Xu, 2021). Considering that single-factor tests can be insensitive, we put all variables into the same common factor (Iverson and Maguire, 2000). It was found that the matching of the model is undesirable (χ^2^/df = 11.586, RMSEA = 0.149, CFI = 0.478, TLI = 0.426, IFI =0.480, SRMR = 0.143; Table 2). Thus, this study’s common method bias was not serious.

**Table 2:** Results of confirmatory factor analysis.

Model	χ^2^	df	χ^2^/df	RMSEA	CFI	TLI	IFI	SRMR
PO, PCB, LMX, KH	628.381	224	2.805	0.062	0.913	0.902	0.914	0.051
PO + PCB, LMX, KH	1070.464	227	4.716	0.089	0.819	0.798	0.820	0.081
PO, PCB + LMX, KH	1140.943	227	5.026	0.092	0.804	0.782	0.805	0.092
PO + LMX, PCB, KH	1076.172	227	4.741	0.089	0.818	0.797	0.819	0.073
PO, PCB, LMX + KH	1465.113	227	6.454	0.107	0.734	0.704	0.736	0.123
PO + PCB + LMX + KH	2664.762	230	11.586	0.149	0.478	0.426	0.480	0.143

+ means the combination of two factors into one factor; PO, perceived overqualification; PCB, psychological contract breach; LMX, leader-member exchange; KH, knowledge hiding.

### Validity test

#### Convergent validity

This study tested the convergent validity of the variables, and Table 3 reports the values of standardized loadings, composite reliability (CR), and average variance extracted (AVE). It suggested that the standardized loadings are > 0.3 and AVE > 0.36 at least (Chin, 1998), CR > 0.5 (Asmelash and Kumar, 2019). And the CR of our study is greater than 0.7 and the AVE is around 0.4 and higher which indicates that the variables involved in this study have good convergent validity.

**Table 3:** The standardized loadings, composite reliability (CR) and average extracted variance (AVE).

Variables	Items	Standardized loadings	CR	AVE
PO	PO1	0.397	0.754	0.392
	PO2	0.528		
	PO3	0.720		
	PO4	0.792		
	PO5	0.613		
PCB	PCB1	0.792	0.896	0.634
	PCB2	0.826		
	PCB3	0.833		
	PCB4	0.761		
	PCB5	0.766		
LMX	LMX1	0.423	0.819	0.437
	LMX2	0.633		
	LMX3	0.634		
	LMX4	0.696		
	LMX5	0.765		
	LMX6	0.754		
KH	KH1	0.646	0.874	0.502
	KH2	0.510		
	KH3	0.705		
	KH4	0.779		
	KH5	0.809		
	KH6	0.774		
	KH7	0.690		

#### Confirmatory factor analysis

To test the construct validity of each factor, combined with AMOS 24.0 (Cesur and Durak-Batigun, 2021), we tested the discriminative validity of PO, PCB, LMX, and KH. Table 2 shows that the fitting effect of the quartet model (χ^2^/df = 2.805, RMSEA = 0.062, CFI = 0.913, TLI = 0.902, IFI =0.914, and SRMR = 0.051) is significantly superior to others, which indicates that variables have good differential validity.

Additionally, we tested the discriminant validity of variables, which means whether the square root of AVE of variable is greater than the correlation coefficient among the variables (Fornell and Larcker, 1981). From Table 4, we can see that AVE square root of each variable was larger than the correlation coefficient between other variables, which proved that the discriminant validity of this study was good.

**Table 4:** Square root of AVE and correlation coefficient.

Variables	PO	PCB	LMX	KH
PO	**0.626**			
PCB	0.293	**0.796**		
LMX	−0.116	−0.497	**0.661**	
KH	0.190	0.291	−0.245	**0.708**

### Descriptive statistical

Table 5 reveals the average value, standard deviation, and internal consistency reliability of this paper’s variables. According to Table 5, PO and PCB (*β* = 0.293, *p* < 0.001), PO and KH (*β* = 0.190, p < 0.001), P and CB has a remarkably active correlation with KH (*β* = 0.291, *p* < 0.001). Moreover, the variance inflation factors (VIF) of the respective variables are between 1 and 2 (all less than 10), and it is generally believed that there is no multicollinearity problem (O’Brien, 2007), which also means further hypothesis testing.

**Table 5:** Means, standard deviations, and correlations (*N* = 475).

Variable	Mean	SD	1	2	3	4	5	6	7	8	9	10
1. Gender	1.63	0.485										
2. Age	3.52	0.951	−0.170^***^									
3. Education	1.23	0.453	0.024	−0.072								
4. Tenure	3.04	1.047	−0.177^***^	0.593^***^	−0.100^*^							
5. Position	1.90	0.805	−0.211^***^	0.314^***^	0.156^**^	0.383^***^						
6. Company nature	1.67	0.470	−0.032	−0.092^*^	−0.253^***^	−0.148^**^	−0.032					
7. PO	3.078	0.866	−0.075	−0.081	−0.040	−0.155^**^	−0.124^**^	0.035	(0.751)			
8. PCB	2.663	0.920	−0.019	−0.059	0.073	−0.196^***^	−0.113^*^	0.003	0.293^***^	(0.895)		
9. LMX	3.455	0.670	−0.023	0.071	−0.072	0.138^**^	0.189^***^	0.028	−0.116^*^	−0.497^***^	(0.814)	
10. KH	2.211	0.801	−0.115^*^	−0.077	0.031	−0.047	−0.083	−0.049	0.190^***^	0.291^***^	−0.245^***^	(0.871)

The numbers in diagonal brackets are the reliability coefficients of each variable.

* *p* < 0.05;

** *p* < 0.01;

*** *p* < 0.001.

### Hypothesis testing

SPSS can perform regression analysis on variables well (Hopkins and Ferguson, 2014), making it one of the most widely used statistical software in academia. In our study, SPSS 25.0 software was used to verify the hypotheses by regression model. Additionally, we used the three-step analysis method (Baron and Kenny, 1986) in the mediation test. Firstly, we tested the relationship between PO and KH and then checked the relationship between PO and PCB. Finally, the relationship between PO through PCB and KH was examined. After standardizing PO, PCB and LMX, the PO square, PCB square, the interaction between PO and LMX, and the interaction between PO square and LMX are constructed.

#### Principal effect test

In Table 6, PO was significantly positively related with KH (Model 4, *β* = 0.171, *p* = 0.000), and the square of PO was passively related to KH (Model 5, *β* = −0.103, *p* = 0.023) when the term of square of PO was added, with a better model explaining (*ΔR^2^* = 0.058, *F* = 4.616, *p* = 0.000), which indicated the reverse U-shape of PO and KH, and **H1** was verified.

**Table 6:** Mediating effect of psychological contract breach (*N* = 475).

Variable	PCB		KH					
Model	Model 1	Model 2	Model 3	Model 4	Model 5	Model 6	Model 7	Model 8
Gender	−0.061	−0.030	−0.151^**^	−0.131^**^	−0.126^**^	−0.133^**^	−0.122^**^	−0.119^**^
Ages	0.093	0.089	−0.077	−0.079	−0.075	−0.103	−0.095	−0.098
Education	0.068	0.078	0.032	0.038	0.040	0.012	0.015	0.020
Tenure	−0.226^***^	−0.185^**^	0.005	0.031	0.033	0.069	0.074	0.080
Position	−0.079	−0.056	−0.099	−0.084	−0.087	−0.077	−0.070	−0.073
Company nature	−0.009	−0.008	−0.055	−0.054	−0.052	−0.052	−0.041	−0.050
PO		0.266^***^		0.171^***^	0.159^**^			0.090
PO^2^					−0.103^*^			−0.100^*^
PCB						0.286^***^	0.327^***^	0.258^***^
PCB^2^							−0.205^***^	
*F*	4.432^***^	9.224^***^	2.824^*^	4.487^***^	4.616^***^	8.451^***^	10.517^***^	7.842^***^
*R^2^*	0.054	0.122	0.035	0.063	0.073	0.112	0.153	0.132
*ΔR^2^*	0.042	0.108	0.023	0.049	0.058	0.099	0.138	0.115

PO^2^ stands for square term of PO, PCB^2^ stands for square term of PCB.

* *p* < 0.05;

** *p* < 0.01;

*** *p* < 0.001.

#### Mediating effect test

We examined the mediating effect of PCB between PO and KH. As shown in Table 6, the PO was significantly positively related with PCB (Model 2, *β* = 0.266, *p* = 0.000) and KH (Model 4, *β* = 0.171, *p* = 0.000), and **H2** was verified. Further observation showed that PCB was dramatically positively related with KH (Model 6, *β* = 0.286, *p* = 0.000), and the square term of PCB was significantly negatively related with KH (Model 7, *β* = −0.205, *p* = 0.000), indicating that there was a reverse U-shaped relation between PCB and KH, and H3 was verified. Meanwhile, it was found that the square term of PO (Model 8, *β* = −0.100, *p* = 0.022) still had a noteworthy effect on KH after commanding PCB, and the coefficient becomes smaller, indicating that PCB had a sectionally mediating effect on the reverse U-shape connection between PO and KH, and H4 was verified.

#### Moderating effect test

To test the moderating effect of LMX on the PO and KH, this paper first standardized the PO and LMX, and constructed the interaction term to exclude the adverse effect of multi-line collinearity. Table 7 reveals that the interaction of PO and LMX had an arresting impact on KH (Model 3, *β* = 0.096, *p* = 0.031), and the interaction term of square term of PO and LMX had a remarkable effect on KH (Model 4, *β* = 0.132, *p* = 0.036) with the significantly increased model fitting coefficient (*ΔR^2^* = 0.113, *F* = 6.497, *p* = 0.000), which indicated that LMX took a moderating influence on the inverted U-shaped connection between PO and KH, and H5 was verified.

**Table 7:** Moderating effects of leader-member exchange (*N* = 475).

Variable	KH			
Model	Model 1	Model 2	Model 3	Model 4
Gender	−0.151^**^	−0.123^**^	−0.124^**^	−0.126^**^
Ages	−0.077	−0.084	−0.072	−0.079
Education	0.032	0.019	0.013	0.013
Tenure	0.005	0.049	0.036	0.042
Position	−0.099	−0.048	−0.040	−0.045
Company nature	−0.055	−0.048	−0.048	−0.055
PO		0.141^**^	0.137^*^	0.150^**^
PO^2^		−0.092^*^	−0.077	−0.081
LMX		−0.215^***^	−0.210^***^	−0.299^***^
PO*LMX			0.096^*^	−0.111^*^
PO^2^*LMX				0.132^*^
*F*	2.824^*^	6.824^***^	6.656^***^	6.497^***^
*R^2^*	0.035	0.117	0.125	0.134
*ΔR^2^*	0.023	0.100	0.107	0.113

PO, perceived overqualification; LMX, leader-member exchange; PO^2^ stands for square term of PO.

* *p* < 0.05;

** *p* < 0.01;

*** *p* < 0.001.

**Figure 2:**
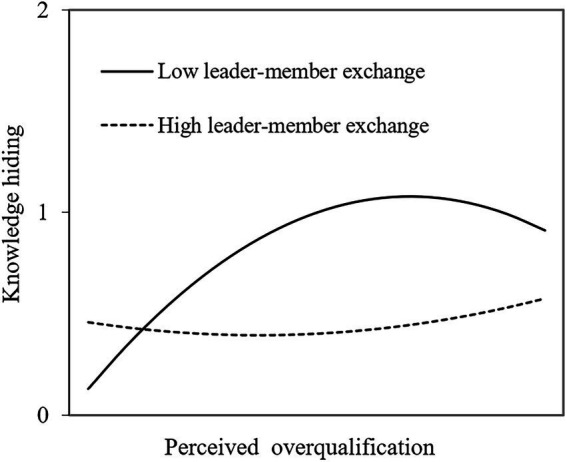
Moderating effect of leader-member exchange.

Secondly, referring to Dawson (2014), the mean value of LMX plus and minus one standard deviation was used to draw the interaction effect graph (see Figure 2). It showed that the moderating influence of LMX on employees’ PO and KH was approximately presented as an inverted U-shaped curve and a slightly upward sloping straight line. Compared with high-quality LMX, the curve was steeper and the slope was larger under low-quality LMX, indicating that the active and inactive relationships between PO and KH were more significant in this case, and H5 was further verified.

## Conclusion and discussion

### Conclusion

This paper inspected the mediator effect of psychological contract breach (PCB) on the reverse U-shaped relation between perceived overqualification (PO) and knowledge hiding (KH), and tested the moderating effect of leader-member exchange (LMX). The results manifested that PO is in a reverse U-shaped relation with KH, and low-middle (middle-high) level of PO positively (negatively) affects KH. Moreover, PCB, which can be promoted by PO, had a reverse U-shaped contact with KH, and also partially mediated the inverted U-shaped connection between PO and KH. LMX moderated the reverse U shaped relation between PO and KH. Compared with high-quality LMX, the positive and negative effects of PO on KH are stronger in terms of low-quality LMX.

### Theoretical implications

Our study contributes to the existing literature in three ways. Firstly, we expanded the study on the relationship between PO and KH. In the study of antecedents of knowledge hiding, PO has not been fully discussed. In terms of study on the relationship between PO and KH, the positive impact of PO on KH (Li et al., 2021) and their U-type non-linear relation (Ma and Zhang, 2021) have been confirmed. However, we confirmed the inverted U-shaped relationship between PO and KH, which is inconsistent with existing studies, revealed that the low-middle level of PO positively promotes KH, and the middle-high level of PO negatively affects KH over a certain critical point. This result provides a new way to explain the relationship between PO and KH, and enriched the related research in the field of PO and knowledge management.

Secondly, previous literature has analyzed the influence mechanism of the PO and KH from the perspective of emotion (Ma and Zhang, 2021), while we find an inverted U-shaped relationship between PCB and KH from the perspective of cognition. And our research result directly responds to previous suggestions for analysis from the perspective of cognition (Li et al., 2021; Ma and Zhang, 2021), and also expands the previous linear relationship between PCB and individual behavior (Shen et al., 2019). Our study shows that PCB does not necessarily promote employees’ negative behaviors, which is consistent with previous views (Solinger et al., 2016). Employees are more likely to hide knowledge under proper PCB, while the employees with lower and higher PCB have less KH, which provides a new explanation and reference for a comprehensive understanding of PCB as a mediator.

Thirdly, there is little research on the boundary conditions of KH of overqualified employees, which our study contributes to by confirming the moderating effect of LMX on the reverse U-shaped relationship between PO and KH. This study confirmed the significant positive correlation between employees’ PO and KH under high-quality LMX. And under low-quality LMX, PO had a more significant negative effect on KH, indicating that low-quality LMX had a stronger effect on the KH of PO, which provides a new perspective to interpret the boundary condition for the relationship between PO and KH.

### Practical implications

There are a large number of employees with excessive qualifications in the workplace (Erdogan et al., 2020). Organizations hope that employees can share knowledge and contribute to organizational innovation, although the reality is not always as organizations expect. Now, it is urgent to discuss how to take full advantage of overqualified employees to provide positive help for organizations’ sustainable development. Our conclusion reveals the double-sided effect of PO on KH, and provides the following management implications for organizations to manage employees with overqualification.

Firstly, this paper reveals an important phenomenon: employees with different levels of PO have different attitudes toward KH, that is, those high-quality talents in the organization do not always hide knowledge, and we are should treat the phenomenon of overqualification rationally. On the one hand, employees’ PO can lead to KH, which generally means that organizations need to focus on the matching of employees’ qualifications and positions. For competent people, excessive training provided by organizations may sometimes bring adverse results (Ma and Zhang, 2021). Managers should consider the promotion channels and career planning of overqualified employees, and try to provide a platform for their value and talent to alleviate their perceived overqualification. On the other hand, although PO is inevitable (Erdogan and Bauer, 2021), it does not necessarily promote employees’ KH. A certain degree of PO can reduce KH and increase the opportunities for knowledge exchange within the organization. For promising organizations, recruiting employees with higher qualifications than their posts may be more beneficial to the long-term and sustainable development of organization [as Erdogan et al., 2011 suggested], and also explains the enterprise management phenomenon that Siemens in Germany requires to recruit employees with “great development potential, at least one or two levels higher than their posts” (Zhuang and Jiang, 2011).

Secondly, managers need to understand the psychological state of employees and reasonably grasp employees’ psychological contract with organizations. On the one hand, managers need to understand and take targeted strategies to repair the psychological tacit with overqualified employees (Henderson et al., 2020), when the degree of PCB is at a low or medium level, such as asking employees’ true thoughts in advance when arranging jobs and making career plans. And it is important for managers to closely observe the psychological state of both employees and their colleagues and regularly organize and launch team-building activities to enhance the relationship and build trust (Bari et al., 2020b) between employees and colleagues. On the other hand, when the degree of PCB is high, managers ought to guide employees to stay consistent with the values of the organization, such as strengthening the construction of team culture, carrying out organizational training, and increasing the interaction between employees and the organization.

Eventually, managers should properly control the distance between themselves and their employees. Managers should pay attention, trust, and support to employees with low or high levels of PO. However, although relevant research affirmed that maintaining a good relation between employees and leaders can bring positive effects for individuals and organizations (Graen and Uhl-Bien, 1995; Martin et al., 2016; Choi et al., 2019), we should also be cautious of the employee who is spoiled to engage in negative behavior that is detrimental to the organization. Managers need to establish their own leadership authority, particularly for overqualified employees.

### Limitations and directions for future research

This paper adopts the method of employee self-evaluation, which inevitably has the problem of common method deviation. In the future, a variety of research methods (such as multi-point questionnaire collection or experimental design) can be considered to increase the scientific nature of the research. Next, we only examined the roles of PCB and LMX. Whether other factors (such as self-efficacy, career loneliness, etc.) affect PO and KH remains to be explored. It remains to be probed whether other theories (such as resource conservation theory, competition, and cooperation theory, etc.) can better account for the non-linear relation between PO and KH. Future research can further explore the relationship from these aspects. Then, although we have found the non-linear relationship between PO and KH, there is still a lack of discussion on the hidden dimension of employee rationalization. Future studies can further analyze the reasonable KH of overqualified employees. Moreover, this paper found that the connection between PCB and its outcome variables is not simply linear, and future studies can test the possible non-linear relationship according to this idea.

## Data availability statement

The original contributions presented in the study are included in the article/Supplementary material, further inquiries can be directed to the corresponding author.

## Author contributions

HQZ performed the research design and outline of the manuscript. LZL contributed to drafting the manuscript and analyzing questionnaire data. AHC collected the data and improved the empirical analysis. XMS revised the draft. All authors contributed to the article and approved the submitted version.

## Funding

This article was financially supported by the the Soft Science Project for Sichuan Science and Technology Agency (grant number 2022JDR0024) and Sichuan Grassroots Public Cultural Service Research Center (grant number JY2019B04).

## Conflict of interest

The authors declare that the research was conducted in the absence of any commercial or financial relationships that could be construed as a potential conflict of interest.

## Publisher’s note

All claims expressed in this article are solely those of the authors and do not necessarily represent those of their affiliated organizations, or those of the publisher, the editors and the reviewers. Any product that may be evaluated in this article, or claim that may be made by its manufacturer, is not guaranteed or endorsed by the publisher.

## References

[ref1] Abdullah M. I. Huang D. C. Ali M. Usman M. (2019). Ethical leadership and knowledge hiding: a moderated mediation model of relational social capital, and instrumental thinking. Front. Psychol. 10:2403. doi: 10.3389/fpsyg.2019.02403, PMID: 31708841 PMC6823209

[ref2] Ampofo E. T. (2020). Do job satisfaction and work engagement mediate the effects of psychological contract breach and abusive supervision on hotel employees’ life satisfaction? J. Hosp. Market. Manag. 30, 282–304. doi: 10.1080/19368623.2020.1817222

[ref3] Anser M. K. Ali M. Usman M. Rana M. L. T. Yousaf Z. (2021). Ethical leadership and knowledge hiding: an intervening and interactional analysis. Serv. Ind. J. 41, 307–329. doi: 10.1080/02642069.2020.1739657

[ref4] Asmelash A. G. Kumar S. (2019). Assessing progress of tourism sustainability: developing and validating sustainability indicators. Tour. Manag. 71, 67–83. doi: 10.1016/j.tourman.2018.09.020

[ref5] Bari M. W. Ghaffar M. Ahmad B. (2020b). Knowledge-hiding behaviors and employees’ silence: mediating role of psychological contract breach. J. Knowl. Manag. 24, 2171–2194. doi: 10.1108/JKM-02-2020-0149

[ref6] Bari M. W. Qurrah tul ain, Abrar M. Fanchen M. (2020a). Employees’ responses to psychological contract breach: The mediating role of organizational cynicism. Econ. Ind. Democr. 43, 810–829. doi: 10.1177/0143831X20958478

[ref7] Baron R. M. Kenny D. A. (1986). The moderator-mediator variable distinction in social psychological research: conceptual, strategic, and statistical considerations. J. Pers. Soc. Psychol. 51, 1173–1182. doi: 10.1037/0022-3514.51.6.1173 3806354

[ref8] Buunk A. P. Gibbons F. X. (2007). Social comparison: The end of a theory and the emergence of a field. Organ. Behav. Hum. Decis. Process. 102, 3–21. doi: 10.1016/j.obhdp.2006.09.007

[ref9] Cesur G. Durak-Batigun A. (2021). A Turkish adaptation of the grief cognitions questionnaire: factor analysis, reliability and validity. Curr. Psychol. 40, 72–80. doi: 10.1007/s12144-018-9983-7

[ref10] Chin W. W. (1998). Issues and opinion on structural equation modeling[J]. MIS Q. 22, 7–16.

[ref11] Chiu S. F. Peng J. C. (2008). The relationship between psychological contract breach and employee deviance: The moderating role of hostile attributional style. J. Vocat. Behav. 73, 426–433. doi: 10.1016/j.jvb.2008.08.006

[ref12] Choi W. Kim S. L. Yun S. (2019). A social exchange perspective of abusive supervision and knowledge sharing: investigating the moderating effects of psychological contract fulfillment and self-enhancement motive. J. Bus. Psychol. 34, 305–319. doi: 10.1007/s10869-018-9542-0

[ref13] Connelly C. E. Zweig D. Webster J. Trougakos J. P. (2012). Knowledge hiding in organizations. J. Organ. Behav. 33, 64–88. doi: 10.1002/job.737

[ref14] Dawson J. F. (2014). Moderation in management research: what, why, when, and how. J. Bus. Psychol. 29, 1–19. doi: 10.1007/s10869-013-9308-7

[ref15] Deng H. Guan Y. J. Wu C. H. Erdogan B. Bauer T. Yao X. (2018). A relational model of perceived Overqualification: the moderating role of interpersonal influence on social acceptance. J. Manage. 44, 3288–3310. doi: 10.1177/0149206316668237

[ref16] Erdogan B. Bauer T. N. (2021). Overqualification at work: A review and synthesis of the literature. Annu. Rev. Organ. Psych. 8, 259–283. doi: 10.1146/annurev-orgpsych-012420-055831

[ref17] Erdogan B. Bauer T. N. Peiro J. M. Truxillo D. M. (2011). Overqualified employees: making the best of a potentially bad situation for individuals and organizations. Ind. Organ. Psychol. 4, 215–232. doi: 10.1111/j.1754-9434.2011.01330.x

[ref18] Erdogan B. Karaeminogullari A. Bauer T. N. Ellis A. M. (2020). Perceived Overqualification at work: implications for extra-role behaviors and advice network centrality. J. Manag. 46, 583–606. doi: 10.1177/0149206318804331

[ref19] Fine S. Edward M. (2017). Breaking the rules, not the law: The potential risks of counterproductive work behaviors among overqualified employees. Int. J. Sel. Assess. 25, 401–405. doi: 10.1111/ijsa.12194

[ref21] Fong P. S. W. Men C. H. Luo J. L. Jia R. Q. (2018). Knowledge hiding and team creativity: the contingent role of task interdependence. Manag. Decis. 56, 329–343. doi: 10.1108/MD-11-2016-0778

[ref22] Fornell C. Larcker D. F. (1981). Evaluating structural equation models with unobservable variables and measurement error[J]. J. Market. Res. 18, 39–50. doi: 10.1177/002224378101800104

[ref23] Gerber J. P. Wheeler L. Suls J. (2018). A social comparison theory Meta-analysis 60+years On. Psychol. Bull. 144, 177–197. doi: 10.1037/bul0000127, PMID: 29144145

[ref24] Ghani U. Teo T. Li Y. Usman M. Islam Z. U. Gul H.. (2020). Tit for tat: abusive supervision and knowledge hiding-The role of psychological contract breach and psychological ownership. Int. J. Environ. Res. Public Health 17:1240. doi: 10.3390/ijerph17041240, PMID: 32075163 PMC7068359

[ref25] Graen G. B. Uhl-Bien M. (1995). Development of leader-member exchange (LMX) theory of leadership over 25 years: applying a multi-level multi-domain perspective. Leadership Q. 6, 219–247. doi: 10.1016/1048-9843(95)90036-5

[ref26] Griep Y. Vantilborgh T. (2018). Reciprocal effects of psychological contract breach on counterproductive and organizational citizenship behaviors: The role of time. J. Vocat. Behav. 104, 141–153. doi: 10.1016/j.jvb.2017.10.013

[ref27] Guo L. M. Cheng K. Luo J. L. Zhao H. D. (2021). Trapped in a loss spiral: how and when work alienation relates to knowledge hiding. Int. J. Hum. Resour. Manag. 1–30. doi: 10.1080/09585192.2021.1937672

[ref28] Gupta V. Agarwal U. A. Khatri N. (2016). The relationships between perceived organizational support, affective commitment, psychological contract breach, organizational citizenship behaviour and work engagement. J. Adv. Nurs. 72, 2806–2817. doi: 10.1111/jan.13043, PMID: 27293180

[ref29] Harari M. B. Manapragada A. Viswesvaran C. (2017). Who thinks they're a big fish in a small pond and why does it matter? A meta-analysis of perceived overqualification. J. Vocat. Behav. 102, 28–47. doi: 10.1016/j.jvb.2017.06.002

[ref30] Hartmann N. N. Rutherford B. N. (2015). Psychological contract breach's antecedents and outcomes in salespeople: The roles of psychological climate, job attitudes, and turnover intention. Ind. Mark. Manage. 51, 158–170. doi: 10.1016/j.indmarman.2015.07.017

[ref31] He P. X. Jiang C. L. Xu Z. X. Shen C. A. (2021). Knowledge hiding: current research status and future research directions. Front. Psychol. 12:748237. doi: 10.3389/fpsyg.2021.748237, PMID: 34777143 PMC8586422

[ref32] Henderson K. E. Welsh E. T. O'Leary-Kelly A. M. (2020). “oops, I did it” or “it Wasn’t me:” An examination of psychological contract breach repair tactics. J. Bus. Psychol. 35, 347–362. doi: 10.1007/s10869-019-09624-z

[ref33] Hopkins L. Ferguson K. E. (2014). Looking forward: The role of multiple regression in family business research. J. Fam. Bus. Strat. 5, 52–62. doi: 10.1016/j.jfbs.2014.01.008

[ref34] Huo W. W. Cai Z. Y. Luo J. L. Men C. H. Jia R. Q. (2016). Antecedents and intervention mechanisms: a multi-level study of R&D team’s knowledge hiding behavior. J. Knowl. Manag. 20, 880–897. doi: 10.1108/JKM-11-2015-0451

[ref35] Iverson R. D. Maguire C. (2000). The relationship between job and life satisfaction: evidence from a remote mining community. Hum. Relat. 53, 807–839. doi: 10.1177/0018726700536003

[ref36] Jackson C. L. Colquitt J. A. Wesson M. J. Zapata-Phelan C. P. (2006). Psychological collectivism: a measurement validation and linkage to group member performance. J. Appl. Psychol. 91, 884–899. doi: 10.1037/0021-9010.91.4.884, PMID: 16834512

[ref37] Jahanzeb S. De Clercq D. Fatima T. (2021). Organizational injustice and knowledge hiding: the roles of organizational dis-identification and benevolence. Manag. Decis. 59, 446–462. doi: 10.1108/MD-05-2019-0581

[ref38] Jha J. K. Varkkey B. (2018). Are you a cistern or a channel? Exploring factors triggering knowledge-hiding behavior at the workplace: evidence from the Indian R&D professionals. J. Knowl. Manag. 22, 824–849. doi: 10.1108/JKM-02-2017-0048

[ref39] Kim S. Y. (2012). Sample size requirements in single-and multiphase growth mixture models: A Monte Carlo simulation study. Struct. Equ. Modeling 19, 457–476. doi: 10.1080/10705511.2012.687672

[ref40] Kim M. S. Phillips J. M. Park W.-W. Gully S. M. (2021). When leader-member exchange leads to knowledge sharing: The roles of generalself-efficacy, team leader modeling, and LMX differentiation. Int. J. Hum. Resour. Manag. 1–28. doi: 10.1080/09585192.2021.1886150

[ref41] Kong M. Xu H. Y. Zhou A. Q. Yuan Y. (2019). Implicit followership theory to employee creativity: The roles of leader-member exchange, self-efficacy and intrinsic motivation. J. Manag. Organ. 25, 81–95. doi: 10.1017/jmo.2017.18

[ref42] Kutaula S. Gillani A. Budhwar P. S. (2020). An analysis of employment relationships in Asia using psychological contract theory: A review and research agenda. Hum. Resour. Manage. Rev. 30:100707. doi: 10.1016/j.hrmr.2019.100707

[ref43] Li S. Chen Y. (2018). The relationship Between psychological contract breach and Employees' counterproductive work behaviors: The mediating effect of organizational cynicism and work alienation. Front. Psychol. 9:1273. doi: 10.3389/fpsyg.2018.01273, PMID: 30100888 PMC6072872

[ref44] Li C. S. Liao H. Y. Han Y. Q. (2021). I despise but also envy you: A dyadic investigation of perceived overqualification, perceived relative qualification, and knowledge hiding. Pers. Psychol. 75, 91–118. doi: 10.1111/peps.12444

[ref45] Lin B. L. Law K. S. Zhou J. (2017). Why is underemployment related to creativity and OCB?A task crafting explanation of the curvilinear moderated relations. Acad. Manage. J. 60, 156–177. doi: 10.5465/amj.2014.0470

[ref46] Liu S. Q. Luksyte A. Zhou L. Shi J. Q. Wang M. (2015). Overqualification and counterproductive work behaviors: examining a moderated mediation model. J. Organ. Behav. 36, 250–271. doi: 10.1002/job.1979

[ref47] Liu S. Wang M. (2012). Perceived Overqualification: A review and recommendations for research and practice. Res. Occup. Stress Wellbeing. 10, 1–42. doi: 10.1108/S1479-3555(2012)0000010005

[ref48] Luksyte A. Spitzmueller C. Maynard D. C. (2011). Why do overqualified incumbents deviate? Examining Multiple Mediators. J. Occup. Health Psychol. 16, 279–296. doi: 10.1037/a0022709, PMID: 21728436

[ref49] Ma C. Lin X. S. Chen Z. X. Wei W. (2020). Linking perceived overqualification with task performance and proactivity? An examination from self-concept-based perspective. J. Bus. Res. 118, 199–209. doi: 10.1016/j.jbusres.2020.06.041

[ref50] Ma B. Liu S. S. Lassleben H. Ma G. (2019). The relationships between job insecurity, psychological contract breach and counterproductive workplace behavior: does employment status matter? Pers. Rev. 48, 595–610. doi: 10.1108/PR-04-2018-0138

[ref51] Ma B. Zhang J. (2021). Are overqualified individuals hiding knowledge: the mediating role of negative emotion state. J. Knowl. Manag. doi: 10.1108/JKM-01-2021-0022

[ref52] Major D. A. Morganson V. J. (2011). Coping With work-family conflict: a leader-member exchange perspective. J. Occup. Health Psychol. 16, 126–138. doi: 10.1037/a0021727, PMID: 21280949

[ref53] Martin R. Guillaume Y. Thomas G. Lee A. Epitropaki O. (2016). Leader-member exchange (LMX) and performance: a meta-analytic review. Pers. Psychol. 69, 67–121. doi: 10.1111/peps.12100

[ref55] Matta F. K. Van Dyne L. (2020). Understanding the disparate behavioral consequences of LMX differentiation: the role of social comparison emotions. Acad. Manage. Rev. 45, 154–180. doi: 10.5465/amr.2016.0264

[ref56] Maynard D. C. Joseph T. A. Maynard A. M. (2006). Underemployment, job attitudes, and turnover intentions. J. Organ. Behav. 27, 509–536. doi: 10.1002/job.389

[ref57] Men C. H. Fong P. S. W. Huo W. W. Zhong J. Jia R. Q. Luo J. L. (2020). Ethical leadership and knowledge hiding: A moderated mediation model of psychological safety and mastery climate. J. Bus. Ethics 166, 461–472. doi: 10.1007/s10551-018-4027-7

[ref58] Morrison E. W. Robinson S. L. (1997). When employees feel betrayed: A model of how psychological contract violation develops. Acad. Manage. Rev. 22, 226–256. doi: 10.2307/259230

[ref59] Mubarak N. Osmadi A. Khan J. Mahdiyar A. Riaz A. (2021). What makes people Hide knowledge? Influence of passive leadership and creative self-efficacy. Front. Psychol. 12:740880. doi: 10.3389/fpsyg.2021.740880, PMID: 34690895 PMC8531077

[ref60] Nadeem M. A. Liu Z. Y. Ghani U. Younis A. Xu Y. (2021). Impact of shared goals on knowledge hiding behavior: the moderating role of trust. Manag. Decis. 59, 1312–1332. doi: 10.1108/MD-09-2019-1197

[ref61] Ng T. W. H. Feldman D. C. Butts M. M. (2014). Psychological contract breaches and employee voice behaviour: The moderating effects of changes in social relationships. Eur. J. Work Organ. Psy. 23, 537–553. doi: 10.1080/1359432X.2013.766394

[ref63] O'Brien R. M. (2007). A caution regarding rules of thumb for variance inflation factors. Qual. Quant. 41, 673–690. doi: 10.1007/s11135-006-9018-6

[ref64] Oubrich M. Hakmaoui A. Benhayoun L. Soilen K. S. Abdulkader B. (2021). Impacts of leadership style, organizational design and HRM practices on knowledge hiding: The indirect roles of organizational justice and competitive work environment. J. Bus. Res. 137, 488–499. doi: 10.1016/j.jbusres.2021.08.045

[ref65] Peng Q. P. Zhong X. Liu S. S. Zhou H. K. Ke N. N. (2021). Job autonomy and knowledge hiding: the moderating roles of leader reward omission and person-supervisor fit. Pers. Rev. doi: 10.1108/PR-03-2020-0133

[ref66] Restubog S. L. D. Bordia P. Tang R. L. Krebs S. A. (2010). Investigating the moderating effects of leader-member exchange in the psychological contract breach-employee performance relationship: A test of two competing perspectives. Brit. J. Manage. 21, 422–437. doi: 10.1111/j.1467-8551.2009.00673.x

[ref67] Robinson S. L. Morrison E. W. (2000). The development of psychological contract breach and violation: a longitudinal study. J. Organ. Behav. 21, 525–546. doi: 10.1002/1099-1379(200008)21:5<525::AID-JOB40>3.0.CO;2-T

[ref68] Sanchez-Cardona I. Vera M. Martinez-Lugo M. Rodriguez-Montalban R. Marrero-Centeno J. (2020). When the job does not fit: The moderating role of job crafting and meaningful work in the relation between employees' perceived overqualification and job boredom. J. Career Assess. 28, 257–276. doi: 10.1177/1069072719857174

[ref69] Sedgwick P. (2014). Non-response bias versus response bias. BMJ. 348:g2573. doi: 10.1136/bmj.g2573

[ref001] Shen Y. M. Schaubroeck J. M. Zhao L. Wu L. (2019). Work group climate and behavioral responses to psychological contract breach. Front. Psychol. 10:67. doi: 10.3389/fpsyg.2019.00067 30778308 PMC6369362

[ref70] Solinger O. N. Hofmans J. Bal P. M. Jansen P. G. W. (2016). Bouncing back from psychological contract breach: how commitment recovers over time. J. Organ. Behav. 37, 494–514. doi: 10.1002/job.2047

[ref71] Straatmann T. Koenigschulte S. Hattrup K. Hamborg K. C. (2020). Analysing mediating effects underlying the relationships between P-O fit, P-J fit, and organisational commitment. Int. J. Hum. Resour. Manag. 31, 1533–1559. doi: 10.1080/09585192.2017.1416652

[ref72] Tufan P. Wendt H. (2020). Organizational identification as a mediator for the effects of psychological contract breaches on organizational citizenship behavior: insights from the perspective of ethnic minority employees. Eur. Manag. J. 38, 179–190. doi: 10.1016/j.emj.2019.07.001

[ref73] Usman M. Javed U. Shoukat A. Bashir N. A. (2021). Does meaningful work reduce cyberloafing? Important roles of affective commitment and leader-member exchange. Behav. Inf. Technol. 40, 206–220. doi: 10.1080/0144929X.2019.1683607

[ref74] van Dijk H. Shantz A. Alfes K. (2020). Welcome to the bright side: why, how, and when overqualification enhances performance. Hum. Resour. Manage. Rev. 30:100688. doi: 10.1016/j.hrmr.2019.04.004

[ref75] Vantilborgh T. Bidee J. Pepermans R. Griep Y. Hofmans J. (2016). Antecedents of psychological contract breach: the role of job demands, job resources, and affect. PLoS One 11:e0154696. doi: 10.1371/journal.pone.0154696, PMID: 27171275 PMC4865204

[ref76] Wang C. Y. Feng J. J. Li X. Z. (2021). Allies or rivals: how abusive supervision influences subordinates' knowledge hiding from colleagues. Manag. Decis. 59, 2827–2847. doi: 10.1108/MD-07-2020-0960

[ref77] Yao Z. Luo J. L. Zhang X. C. (2020). Gossip is a fearful thing: the impact of negative workplace gossip on knowledge hiding. J. Knowl. Manag. 24, 1755–1775. doi: 10.1108/JKM-04-2020-0264

[ref78] Yao M. H. Xu Y. J. (2021). Method Bias mechanisms and procedural remedies. Sociol. Methods Res. 1–44. doi: 10.1177/00491241211043141

[ref79] Yu H. P. Yang F. Wang T. Sun J. M. Hu W. J. (2019). How perceived overqualification relates to work alienation and emotional exhaustion: the moderating role of LMX. Curr. Psychol. 40, 6067–6075. doi: 10.1007/s12144-019-00538-w

[ref80] Zhang Z. Min M. (2021). Organizational rewards and knowledge hiding: task attributes as contingencies. Manag. Decis. 59, 2385–2404. doi: 10.1108/MD-02-2020-0150

[ref81] Zhang M. Wang F. Li N. (2021b). The effect of perceived Overqualification on creative performance: person-organization fit perspective. Front. Psychol. 12:582367. doi: 10.3389/fpsyg.2021.582367, PMID: 34054629 PMC8155303

[ref82] Zhang F. Wang B. Qian J. Parker S. (2021a). Job crafting towards strengths and job crafting towards interests in overqualified employees: different outcomes and boundary effects. J. Organ. Behav. 42, 587–603. doi: 10.1002/job.2517

[ref83] Zhao H. D. Liu W. W. Li J. Yu X. Y. (2019). Leader-member exchange, organizational identification, and knowledge hiding: The moderating role of relative leader-member exchange. J. Organ. Behav. 40, 834–848. doi: 10.1002/job.2359

[ref84] Zhao H. D. Xia Q. He P. X. Sheard G. Wan P. (2016). Workplace ostracism and knowledge hiding in service organizations. Int. J. Hosp. Manag. 59, 84–94. doi: 10.1016/j.ijhm.2016.09.009

[ref85] Zhuang J. Jiang D. (2011). On cross-cultural management of human resources -- A case study of Siemens LTD. Natl. Bus. Situat. (Theoret. Res.), 29–30. doi: 10.16834/j.cnki.issn1009-5292.2011.01.012

